# Supplementary material for: A Biocontrol Strain of Pseudomonas aeruginosa CQ-40 Promote Growth and Control Botrytis cinerea in Tomato
Source: Pathogens. 2020 Dec 31;10(1):22. doi: 10.3390/pathogens10010022 (PMC7824093; doi:10.3390/pathogens10010022)
Supplement: Supplementary file 1 [file pathogens-10-00022-s001.pdf]

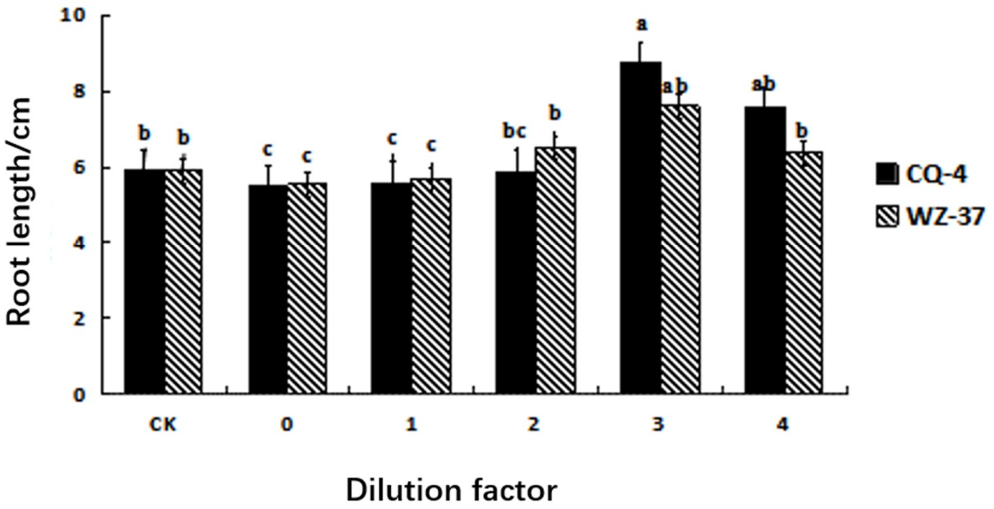

Figure S1. Effect of different dilutions of bacterial suspension on the growth of tomato seed radicle.

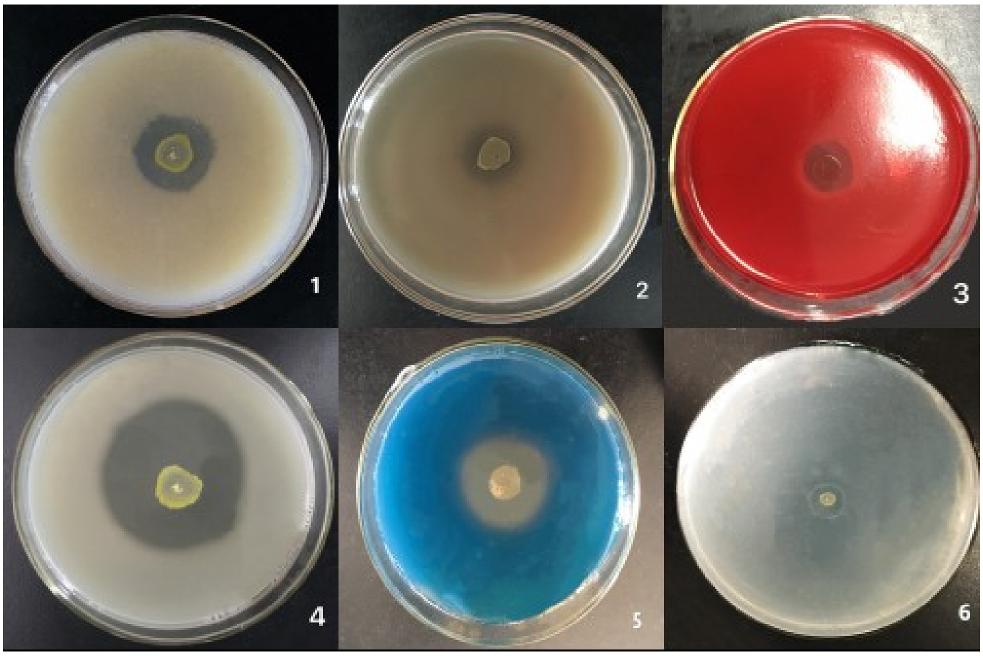

Figure S2. Detection of secretory substance in strains CQ-4.

Note: 1: Inorganic phosphorus 2: Organic phosphorus 3: Cellulase 4: Protease 5: Siderophore 6: Nitrogen fixation

Table S1. Physiological and biochemical characteristics of CQ-4.

Identification index	Strain CQ-4
Fluorescence	Yellow green pigment
Salt tolerance	≤5%
gram stain	G-
Glucose oxidation / fermentation（O/F）	O
Starch hydrolysis	+
Catalase test	+
Aerobic or anaerobic	Aerobic
Nitrate reduction reaction	+
Gelatin liquefaction	+
Citrate reaction	+
Urease reaction	+
Methyl red reaction	-
V.P.	-
H2S	-
Indole	-

Table S2. Antibacterial spectrum of biocontrol bacteria CQ-4.

Pathogenic bacteria	Inhibition zone width /mm	
	CQ-4	WZ-37
Botrytis cinerea	9.6±0.3ab	8.1±0.1ab
Fusarium bulbigenum var. lycopersici	10.8±0.2ab	7.3±0.1bc
Fusarium oxysporum f.sp. niveum	9.3±0.1bc	6.5±0.2cd
Fusariumoxysporum（Schl.）f.sp.	10.3±0.6a	5.2±0.3e
Fusarium oxysporum f. sp.	7.0±0.3c	5.3±0.2e
Pythiumsp.	7.2±0.4c	5.2±0.3e
Sclerotinia sclerotiorum（Lib.）de Bary	8.5±0.3bc	9.0±0.4a

Fusarium graminearum	5.2±0.1e	8.3±0.3ab
Fusarium sp.	4.0±0.2e	8.5±0.2a
Colletotrichum lagenarium	7.0±0.1c	6.0±0.2cd
Bean anthracnose	8.0±0.1bcd	5.8±0.1d
Alternaria spp	6.2±0.3de	5.5±0.1de
Fusarium oxysporium Schelcht	9.03±0.3bc	7.0±0.1c
F.equiseti	7.5±0.2cd	8.0±0.5abc
Fusarium verticillioides	7.6±0.3cd	6.0±0.3cd

Table S3. Effects of biocontrol bacteriaon on the in vitro leaves of tomato gray mold.

treatment	Incidence /%	Disease index	Prevention effect /%
Pyrimethanil	20.17cd	14.07b	76.47a
CQ-4	33.24b	17.03c	71.52c
WZ-37	21.35c	15.33b	74.36b
CK	93.33a	59.81a	-

Table S4. Effects of biocontrol bacteriaon on the in vitro fruits of tomato gray mold.

treatment	Incidence /%	Disease index	Prevention effect /%
Pyrimethanil	16.67c	13.33b	64.71ab
WZ-37	20.43d	15.78c	58.23b
CQ-4	15.58b	12.85b	65.98a
CK	53.33a	37.78a	-

Table S5. The determination of antagonistic activity by biocontrol bacteria.

Identification index	CQ-4	WZ-37
Secret organophosphate	+	-
Secretion of inorganic phosphorus	+	-
Nitrogen Fixation Ability	+	+
Protease	+	-
Cellulase	+	+
Chitinase	-	-
Glucanase	-	-
Siderophore	+	+
HCN	-	-
